# Exposure to Commercial Cigarette Smoke Produces Psychomotor Sensitization via Hyperstimulation of Glutamate Response in the Dorsal Striatum

**DOI:** 10.3390/brainsci11010014

**Published:** 2020-12-24

**Authors:** In Soo Ryu, Jieun Kim, Ju Hwan Yang, Su Yeon Seo, Sumin Sohn, Sunghyun Kim, Kyuhong Lee, Joung-Wook Seo, Eun Sang Choe

**Affiliations:** 1 Department of Biological Sciences, Pusan National University, 63-2 Busandaehak-ro, Geumjeong-gu, Busan 46241, Korea; jieun0479@pusan.ac.kr (J.K.); juhwanyang@pusan.ac.kr (J.H.Y.); ssy1025@kiom.re.kr (S.Y.S.); soomin@pusan.ac.kr (S.S.); s.hyun@pusan.ac.kr (S.K.); 2 Research Center for Convergence Toxicology, Korea Institute of Toxicology, 141 Gajeong-ro, Yuseong-gu, Daejeon 34114, Korea; jwseo@kitox.re.kr; 3 Korean Medicine Fundamental Research Division, Korea Institute of Oriental Medicine, 1672 Yuseong-daero, Yuseong-gu, Daejeon 34054, Korea; 4 Inhalation Toxicology Research Center, Korea Institute of Toxicology, 30 Baekhak 1-gil, Jeongeup 56212, Korea; khlee@kitox.re.kr

**Keywords:** cigarette smoke condensate, nicotine, non-nicotine alkaloid, behavioral sensitization, glutamate

## Abstract

Cigarette smoke is a highly complex mixture of nicotine and non-nicotine constituents. Exposure to cigarette smoke enhances tobacco dependence by potentiating glutamatergic neurotransmission via stimulation of nicotinic acetylcholine receptors (nAChRs). We investigated the effects of nicotine and non-nicotine alkaloids in the cigarette smoke condensates extracted from two commercial cigarette brands in South Korea (KCSC A and KCSC B) on psychomotor behaviors and glutamate levels in the dorsal striatum. Repeated and challenge administration of KCSCs (nicotine content: 0.4 mg/kg, subcutaneous) increased psychomotor behaviors (ambulatory, rearing, and rotational activities) and time spent in psychoactive behavioral states compared to exposure to nicotine (0.4 mg/kg) alone. The increase in psychomotor behaviors lasted longer when exposed to repeated and challenge administration of KCSCs compared to nicotine alone. In parallel with sustained increase in psychomotor behaviors, repeated administration of KCSCs also caused long-lasting glutamate release in the dorsal striatum compared to nicotine alone. KCSC-induced changes in psychomotor behaviors and glutamate levels in the dorsal striatum were found to be strongly correlated. These findings suggest that non-nicotine alkaloids in commercial cigarette smoke synergistically act with nicotine on nAChRs, thereby upregulating glutamatergic response in the dorsal striatum, which contributes to the hypersensitization of psychomotor behaviors.

## 1. Introduction

Nicotine is a major psychoactive alkaloid in tobacco plants and causes dependence due to its rewarding and reinforcing effects [1,2,3,4]. Repeated exposure to nicotine increases dopamine and glutamate releases in the nerve terminals of the dorsal striatum, and nucleus accumbens (NAc) by stimulating excitatory nicotinic acetylcholine receptors (nAChRs) [5,6,7,8,9]. Tobacco plants and products, such as cigarettes and chewing tobacco, contain nicotine as well as non-nicotine alkaloids such as nornicotine, cotinine, and anatabine [10,11,12,13]. These non-nicotine alkaloids can also bind strongly to nAChRs due to their structural similarities with nicotine [14,15], and thus, contribute to development and expression of tobacco dependence [16,17,18,19,20,21]. In addition, acetaldehyde, a component of cigarette smoke, also has psychoactive properties and plays a role in development of tobacco dependence [22,23]. Although tobacco contains various psychoactive substances as well as nicotine, most tobacco dependence studies have been conducted using nicotine only [16,17,18]. Additionally, it is difficult to understand tobacco dependence induced by exposure to commercial cigarettes itself since the nicotine and non-nicotine alkaloid compositions of commercial cigarettes differ significantly from brand to brand [13].

Glutamate is a major excitatory neurotransmitter in the mammalian brain and plays important roles in the development of drug dependence, including dependence on nicotine and tobacco [24,25,26]. Exposure to nicotine increases glutamate release in the dorsal striatum, NAc, ventral tegmental area, and prefrontal cortex by stimulating nAChRs [6,8,25,27,28]. However, repeated administration of 3R4F Kentucky reference cigarette smoke condensate (CSC) causes more prolonged and greater glutamate release in the dorsal striatum compared to nicotine alone [29]. These findings suggest that nicotine and non-nicotine alkaloids in cigarette smoke synergistically upregulate glutamatergic response in the brain reward system by hyperstimulation of nAChRs.

Intermittent and repeated exposure to drugs leads to psychomotor sensitization and hypersensitive motivational behaviors [8,30]. Growing evidence shows that repeated administration of psychoactive drugs, such as nicotine, causes behavioral sensitization by increasing glutamate release in the dorsal striatum and NAc [6,8]. In our previous study, repeated administration of CSC produced more prolonged increase in glutamate release of the dorsal striatum and more hypersensitization of psychomotor behaviors compared to nicotine alone, which are highly correlated [29]. These findings suggest that altered glutamate concentrations in the striatum due to response to the combined effects of nicotine and non-nicotine compounds in cigarette smoke contribute to the hypersensitization of psychomotor activities. However, the relationship between commercial cigarette-induced psychomotor behaviors and hyperactivation of glutamate response is not fully characterized. In this study, we investigated the effects of CSC extracted from two commercial cigarettes in South Korea on psychomotor sensitization and glutamate release in the dorsal striatum that involves in the habitual behavior of drug dependence in rats. In addition, we explored a relapse potential of commercial cigarettes to evoke behavioral sensitization after CSC abstinence in rats.

## 2. Materials and Methods

### 2.1. Animals

A total of 48 adult male Sprague-Dawley rats were used in this study: 24 for behavioral sensitization test and 24 for glutamate biosensing test. Rats weighing between 200 and 230 g (6 weeks old) were purchased from Hyo-Chang Science Co. (Daegu, South Korea). Rats were separated into pairs and acclimated to animal cages for a minimum of 5 days. Food and water were provided *ad libitum*. Animals were maintained under a 12-h light-dark cycle (light on at 8:00 AM) at 21–23 °C and 45–55% relative humidity throughout all experiments. Experimental treatments were applied in a quiet room to minimize environmental stress. All animal procedures were approved by the Institutional Animal Care and Use Committee of Pusan National University (Approval No. PNU-2019-2336, 07-29-2019) and conducted in accordance with the provisions of the Guide for the Care and Use of Laboratory Animals issued by the US National Institute of Health.

### 2.2. Drugs

Nicotine hydrogen tartrate was purchased from Sigma-Aldrich (St. Louis, MO, USA), dissolved in vehicle solution, 1% dimethyl sulfoxide (DMSO)/0.9% physiological saline, and adjusted to pH 7.2–7.4 with sodium hydroxide. Commercial CSCs (KCSC A and KCSC B) were extracted from randomly selected two brands of most preferred cigarettes in South Korea. _L_-glutamic acid (Sigma-Aldrich) and _L_-ascorbic acid (Duchefa Biochemie B.V., Haarlem, Netherlands) were dissolved in phosphate buffered saline (PBS, pH 7.4) to produce glutamate standard and interfering solutions, respectively, for in vitro calibration of glutamate biosensors. All drug solutions were prepared immediately prior to experiments.

### 2.3. Preparation of KCSCs

KCSCs (KCSC A and KCSC B) were prepared by the Research Center for Inhalation Toxicology at the Korea Institute of Toxicology (Jeongeup, South Korea), as previously described [31,32]. Commercial cigarettes were conditioned for a minimum of 72-h prior to use at 21–23 °C and 57–63% relative humidity in accordance with ISO 3402 [33]. KCSCs were generated using a 30-port smoking machine in accordance with ISO 3308 (puff volume 35 mL, drawn over 2 s, time between puffs 60 s, and no vent blocking) [34]. All cigarettes were smoked 3 mm from the filter-tip paper according to ISO 4387 [35]. KCSCs were prepared using a Cambridge filter pad (44 mm; Whatman, Maidstone, UK) after shaking for 30 min, such that the final concentration of total particulate matter was 20 mg/mL in methanol. The methanol was then moved to a vacuum oven for one day. Obtained dried KCSCs were dissolved in the vehicle solution, passed through 0.45 μm polyetrafluoroethylene sterile filters (ThermoFisher Scientific, Waltham, MA, USA), and stored at −80 °C until use. Since it is impossible to analyze all components of KCSCs due to technical difficulties, only the composition of nicotine and seven non-nicotine alkaloids in the KCSCs were analyzed. The contents of nicotine and non-nicotine alkaloids in the two KCSCs are presented in Table 1. Aldehydes were rarely detected in the particulate phase of cigarette smoke than gas phase [36,37,38] due to its volatile property, and thus the possibility of psychoactive effect of aldehydes (e.g., acetaldehyde) in the particulate KCSCs was excluded in this study.

### 2.4. Administration of Nicotine and KCSCs

The rats were given vehicle, nicotine, or KCSCs administration via subcutaneous route (s.c.) once a day. The dose of nicotine was 0.4 mg/kg (1.0 mL/kg), as determined by previous studies [8,29,39]. The extracted KCSCs were dissolved in vehicle solution and diluted to produce working solutions with a nicotine content of 0.4 mg/mL. The vehicle and nicotine groups were used as negative and positive controls, respectively.

### 2.5. Experimental Designs

Two separate experiments were conducted to determine the relationship between KCSC-induced behavioral sensitization and hyperactivation of glutamate response in the dorsal striatum. The first experiment was conducted to determine whether (1) repeated administration of KCSCs for 14 days or (2) challenge administration of KSCSs after withdrawal period alters the behavioral activities. An open-field test was conducted for 60 min after repeated or challenge administration of KCSCs. Rats were randomly divided into four different groups: (1) 14 days repeated vehicle treatment + 6 days withdrawal + vehicle challenge group; (2) 14 days repeated nicotine treatment + 6 days withdrawal + nicotine challenge group; (3) 14 days repeated KCSC A treatment + 6 days withdrawal + KCSC A challenge group; and (4) 14 days repeated KCSC B treatment + 6 days withdrawal + KCSC B challenge group. The detailed timeline is illustrated in Supplementary Figure S1A.

The second experiment was performed to determine whether repeated administration of KCSCs for 14 days alters the extracellular glutamate concentration in the dorsal striatum. We performed real-time glutamate biosensing for 60 min after repeated administration of KCSCs for 14 days. Another cohort of rats was randomly divided into four different groups: (1) 14 days repeated administration of vehicle; (2) 14 days repeated administration of nicotine; (3) 14 days repeated administration of KSCS A; and (4) 14 days repeated administration of KCSC B. The detailed timeline is illustrated in Supplementary Figure S1B.

### 2.6. Open-Field Test

An open-field test was performed as previously described [8,29]. Ambulatory activity (total distance traveled, as determined by horizontal beam breaks), rearing (number of vertical beam breaks), and rotation (number of turns clockwise or counterclockwise) were measured using the infrared photocell-based automated Opto-Varimex-4 Auto-Track system (Columbus Instruments, Columbus, OH, USA) under open-field, illuminated, and sound-attenuated conditions. Rats were acclimated to a behavioral test chamber (44.5 cm × 44.5 cm × 24 cm) for at least 6 days to avoid environmental variations prior to experiments. Three pairs of sensors were positioned on x-, y- (horizontal), and z- (vertical, placed above normal animal height) axes to provide coordinates that recognizes ambulatory and rearing activities. Each sensor pair produced 16 infrared light beams in the test chamber (beam scan rate = 10 Hz). The Auto-Track system senses the presence of animals using infrared beam interruptions. Changes in ambulatory activity (cm), rearing (count), and rotation (count) activities were measured in 1 min intervals between 30 min before and 60 min after drug administration. Data were transferred from all sensors to a computer running Opto-Varimex 4 Auto Track Rapid Release software (software version 4.99B, Columbus Instruments).

### 2.7. Surgery for Real-Time Glutamate Biosensing

Brain surgery for glutamate biosensing was performed as previously described [8,29]. Briefly, rats were anesthetized with a mixture of Zoletil 50 (tiletamine, 18.75 mg/kg) (Virbac Korea, Seoul, South Korea) and Rompun (xylazine, 5.8 mg/kg) (Bayer Korea, Seoul, South Korea) by intraperitoneal (i.p.) injection and placed in a stereotaxic apparatus. Under aseptic conditions, a BASi Rat Guide Cannula (Pinnacle Technology, Lawrence, KS, USA) (inner diameter, 0.7 mm; length, 10 mm) was surgically implanted into the center of the right dorsal striatum (1.0 mm anterior to bregma, 2.5 mm right of midline, and 5 mm below the skull surface) to allow insertion of a glutamate biosensor. BLE Rat Hat Bottoms (Pinnacle Technology) were used to enable potentiostat placement (Pinnacle Technology) and were covered with BLE Rat Hat Tops (Pinnacle Technology). After surgery, rats were given a minimum of 6 days of recovery in home cages and treated with 0.1 mL of gentamycin (i.p., Eagle Vet, Seoul, South Korea) prior to the first administration of the vehicle, nicotine, KCSC A, and KCSC B. Physical accuracies of cannula implantations were verified by reconstructing guide cannula placements after glutamate biosensing (Supplementary Figure S2). The presence of guide cannula-induced gliosis and the placement of correct glutamate biosensor insertion were verified by Nissl staining (data not shown).

### 2.8. In Vitro Calibration and In Vivo Real-Time Glutamate Biosensing

Real-time glutamate biosensing was performed as previously described [8] using commercial _L_-glutamate oxidase-based glutamate biosensors (glutamate biosensors) (Pinnacle Technology) and _L_-glutamate oxidase-free glutamate biosensors (glutamate null biosensors) (Pinnacle Technology). Before and after measurements, calibrations were conducted in PBS (pH 7.4) by gradually increasing glutamate concentrations from 0 to 4 μM in 1 μM increments. A single addition of 250 μM ascorbic acid, which commonly causes biological interference, did not interfere with glutamate detection (data not shown), which concurs with the results of our previous study on glutamate biosensors [8]. All calibration procedures were performed at 37 °C and a minimum of 5 min was allowed prior to measurements to ensure conditions had stabilized because biosensor sensitivity is directly influenced by temperature [40]. In addition, since glutamate biosensor outputs are inversely related to changes in currents in vivo, rats were acclimated to testing environments for a minimum of 120 min after biosensor insertion. When currents had stabilized, real-time glutamate biosensing in the dorsal striatum was conducted in freely moving rats for 60 min after the final administration of vehicle, nicotine, KCSC A, and KCSC B in home cages. Because the baseline currents of individual rats were slightly different, we adjusted absolute currents induced by the administration of vehicle, nicotine, KCSC A, and KCSC B by setting basal values at 0 nA. Changes of glutamate currents in the dorsal striatum were then converted into changes in glutamate concentrations using individual sensor calibration plots. Data were sampled at 1 Hz using SIRENIA acquisition software (version 1.6.1, Pinnacle Technology).

### 2.9. Statistics

Data are represented as mean ± standard error of the mean (SEM). Bonferroni’s post-hoc test was used for all repeated measures followed by two-way analysis of variance (ANOVA) and multiple comparison test. Analysis was conducted using GraphPad Prism 8 (GraphPad Software, La Jolla, CA, USA). Statistical significance was accepted for *p* values of < 0.05.

## 3. Results

### 3.1. Repeated Administration of KCSCs Increased Ambulatory, Rearing, and Rotational Activities

**Figure 1:**
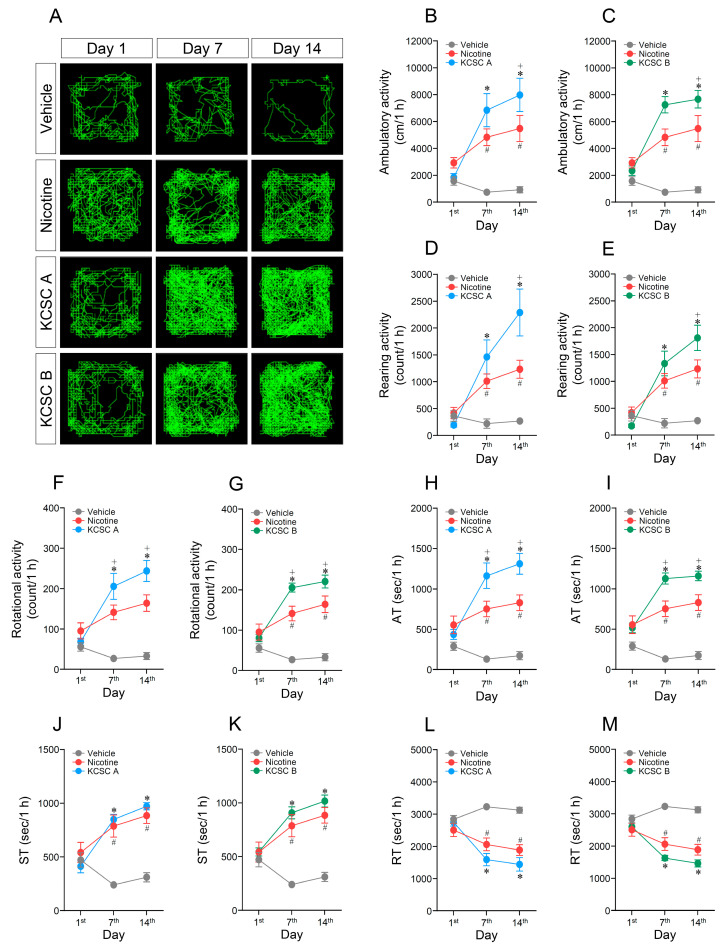
Changes in behavioral activities after acute or repeated administration of two cigarette smoke condensates extracted from two commercial cigarette brands in South Korea (KCSC A and KCSC B). Statistical significance was determined by two-way RM-ANOVA and multiple comparison test with Bonferroni’s post-hoc test. ^#^ *p* < 0.05, repeated vehicle group vs. repeated nicotine group; * *p* < 0.05, repeated vehicle group vs. repeated KCSC groups; ^+^ *p* < 0.05, repeated nicotine group vs. repeated KCSC groups. AT, ambulatory time; ST, stereotypy time; RT, resting time. *n* = 6 per group.

Since repeated exposure to 3R4F CSC (extracted from 3R4F Kentucky Reference cigarette) increased psychomotor activities compared to repeated treatment of nicotine alone [29], an open-field test using two different KCSCs was performed to determine whether repeated exposure to commercial cigarettes alters psychomotor activities such as ambulatory, rearing, and rotational activities. The results showed that repeated administration of KCSC A increased ambulatory activity (two-way RM-ANOVA, Time: F_(2, 45)_ = 11.01, *p* < 0.05; Treatment: F_(2, 45)_ = 30.71, *p* < 0.05; Time × Treatment: F_(4, 45)_ = 6.37, *p* < 0.05) (multiple comparison test, 7th day: F_(2, 15)_ = 15.16, *p* < 0.05; 14th day: F_(2, 15)_ = 15.22, *p* < 0.05) (Figure 1A,B) compared to the vehicle and nicotine control groups. Similarly, repeated administration of KCSC B also increased ambulatory activity (two-way RM-ANOVA, Time: F_(2, 45)_ = 61.19, *p* < 0.05; Treatment: F_(4, 45)_ = 10.14, *p* < 0.05; Time × Treatment: F_(2, 45)_ = 17.50, *p* < 0.05) (multiple comparison test, 7th day: F_(2, 15)_ = 42.21, *p* < 0.05; 14th day: F_(2, 15)_ = 25.15, *p* < 0.05) (Figure 1A,C) compared to the vehicle and nicotine control groups. However, acute administration of the vehicle, nicotine, KCSC A, and KCSC B did not alter ambulatory activity (Figure 1A–C).

Repeated KCSC A administration increased rearing activity (two-way RM-ANOVA, Time: F_(2, 45)_ = 16.27, *p* < 0.05; Treatment: F_(2, 45)_ = 19.52, *p* < 0.05; Time × Treatment: F_(4, 45)_ = 7.58, *p* < 0.05) (multiple comparison test, 7th day: F_(2, 15)_ = 9.31, *p* < 0.05; 14th day: F_(2, 15)_ = 13.85, *p* < 0.05) (Figure 1D), and rotational activity (two-way RM-ANOVA, Time: F_(2, 45)_ = 12.38, *p* < 0.05; Treatment: F_(2, 45)_ = 41.08, *p* < 0.05; Time × Treatment: F_(4, 45)_ = 8.17, *p* < 0.05) (multiple comparison test, 7th day: F_(2, 15)_ = 17.91, *p* < 0.05; 14th day: F_(2, 15)_ = 29.18, *p* < 0.05) (Figure 1F) compared to the vehicle and nicotine control groups. Similar to KCSC A, repeated administration of KCSC B also increased rearing activity (two-way RM-ANOVA, Time: F_(2, 45)_ = 23.22, *p* < 0.05; Treatment: F_(2, 45)_ = 26.09, *p* < 0.05; Time × Treatment: F_(4, 45)_ = 9.79, *p* < 0.05) (multiple comparison test, 7th day: F_(2, 15)_ = 12.41, *p* < 0.05; 14th day: F_(2, 15)_ = 21.17, *p* < 0.05) (Figure 1E) and rotational activity (two-way RM-ANOVA, Time: F_(2, 45)_ = 15.73, *p* < 0.05; Treatment: F_(2, 45)_ = 68.08, *p* < 0.05; Time × Treatment: F_(4, 45)_ = 10.50, *p* < 0.05) (multiple comparison test, 7th day: F_(2, 15)_ = 52.87, *p* < 0.05; 14th day: F_(2, 15)_ = 36.92, *p* < 0.05) (Figure 1G) compared to vehicle and nicotine control groups. However, there was no difference in rearing and rotational activities among the acute administration of vehicle, nicotine, KCSC A, and KCSC B groups (Figure 1D–G).

### 3.2. Repeated Administration of KCSCs Increased Ambulatory and Stereotypy Time, but Decreased Resting Time in Behavioral States

In order to analyze the relative proportion of time spent in psychomotor behaviors increased by repeated KCSCs administration, we analyzed time spent in ambulatory, stereotypy, and resting behavioral states. The results revealed that repeated administration of KCSC A increased ambulatory time (two-way RM-ANOVA, Time: F_(2, 45)_ = 10.56, *p* < 0.05; Treatment: F_(2, 45)_ = 51.18, *p* < 0.05; Time × Treatment: F_(4, 45)_ = 8.32, *p* < 0.05) (multiple comparison test, 7th day: F_(2, 15)_ = 23.46, *p* < 0.05; 14th day: F_(2, 15)_ = 34.46, *p* < 0.05) compared to the vehicle and nicotine control groups (Figure 1H). Similarly, repeated administration of KCSC B also increased ambulatory time (two-way RM-ANOVA, Time: F_(2, 45)_ = 11.27, *p* < 0.05; Treatment: F_(2, 45)_ = 79.98, *p* < 0.05; Time × Treatment: F_(4, 45)_ = 9.18, *p* < 0.05) (multiple comparison test, 7th day: F_(2, 15)_ = 52.44, *p* < 0.05; 14th day: F_(2, 15)_ = 49.32, *p* < 0.05) compared to vehicle or nicotine control groups (Figure 1I). Time spent in stereotypy behaviors was also increased after repeated administration of KCSC A (two-way RM-ANOVA, Time: F_(2, 45)_ = 10.80, *p* < 0.05; Treatment: F_(2, 45)_ = 37.39, *p* < 0.05; Time × Treatment: F_(4, 45)_ = 9.90, *p* < 0.05) (multiple comparison test, 7th day: F_(2, 15)_ = 25.19, *p* < 0.05; 14th day: F_(2, 15)_ = 47.19, *p* < 0.05) and KCSC B (two-way RM-ANOVA, Time: F_(2, 45)_ = 11.27, *p* < 0.05; Treatment: F_(2, 45)_ = 79.98, *p* < 0.05; Time × Treatment: F_(4, 45)_ = 9.18, *p* < 0.05) (multiple comparison test, 7th day: F_(2, 15)_ = 26.59, *p* < 0.05; 14th day: F_(2, 15)_ = 41.23, *p* < 0.05) compared to the vehicle control group (Figure 1J,K). In contrast, time spent in resting behaviors was decreased after repeated administration of KCSC A (two-way RM-ANOVA, Time: F_(2, 45)_ = 9.68, *p* < 0.05; Treatment: F_(2, 45)_ = 43.59, *p* < 0.05; Time × Treatment: F_(4, 45)_ = 8.31, *p* < 0.05) (multiple comparison test, 7th day: F_(2, 15)_ = 27.81, *p* < 0.05; 14th day: F_(2, 15)_ = 28.84, *p* < 0.05) and KCSC B (two-way RM-ANOVA, Time: F_(2, 45)_ = 10.51, *p* < 0.05; Treatment: F_(2, 45)_ = 62.98, *p* < 0.05; Time × Treatment: F_(4, 45)_ = 9.18, *p* < 0.05) (multiple comparison test, 7th day: F_(2, 15)_ = 43.18, *p* < 0.05; 14th day: F_(2, 15)_ = 46.59, *p* < 0.05) compared to the vehicle control group (Figure 1L,M). Relative ratios of ambulatory, stereotypy, and resting times (ambulatory time: stereotypy time: resting time) in the behaviors on the 14th day were as following: (1) Vehicle (4.77:8.63:86.60); (2) nicotine (23.06:24.58:52.36); (3) KCSC A (36.32:26.89:36.79); (4) KCSC B (32.21:27.21:40.58) (Supplementary Figure S3).

### 3.3. Repeated Administration of KCSCs Induced Prolonged Increases in Ambulatory, Rearing, and Rotational Activities

**Figure 2:**
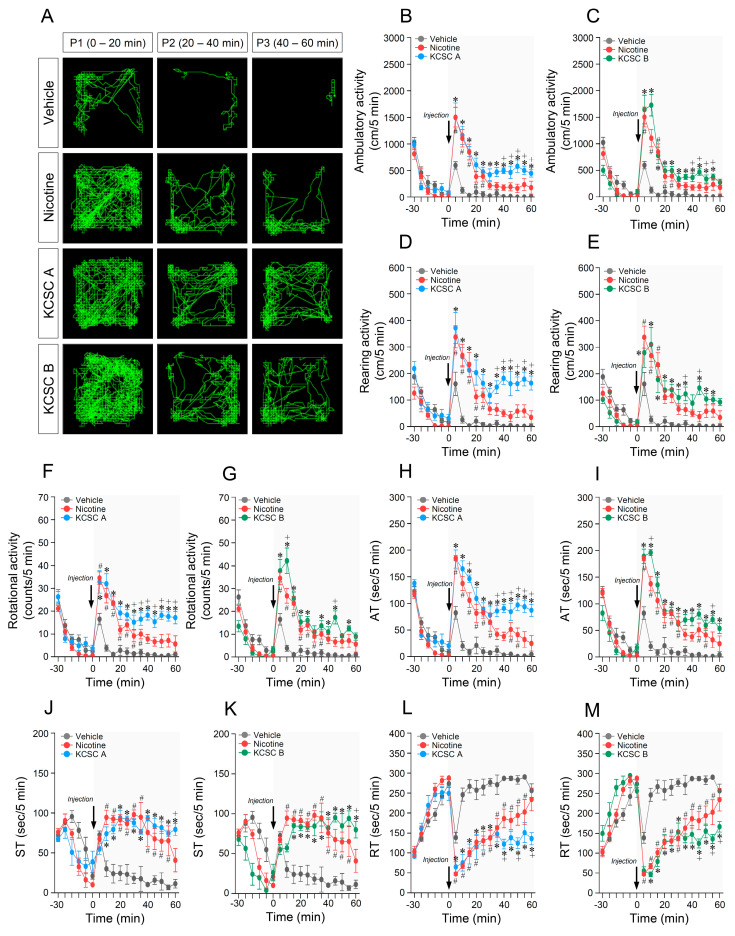
Prolonged changes in behavioral activities after repeated administration of two KCSCs for 14 days. Statistical significance was determined by two-way RM-ANOVA and multiple comparison test with Bonferroni’s post-hoc test. ^#^ *p* < 0.05, repeated vehicle group vs. repeated nicotine group; * *p* < 0.05, repeated vehicle group vs. repeated KCSC groups; ^+^ *p* < 0.05, repeated nicotine group vs. repeated KCSC groups. AT, ambulatory time; ST, stereotypy time; RT, resting time. *n* = 6 per group.

Since repeated administration of 3R4F CSC induced prolonged increases in psychomotor behavioral changes [29], the increase in psychomotor behaviors (ambulatory, rearing, and rotational activities) induced by repeated KCSC administration was divided into three different time periods (P1, 0–20 min; P2, 20–40 min; P3, 40–60 min) and was further analyzed. The results showed that repeated administration of KCSC A produced prolonged increases in ambulatory activity (two-way RM-ANOVA, Time: F_(12, 60)_ = 50.02, *p* < 0.05; Treatment: F_(2, 10)_ = 17.37, *p* < 0.05; Time × Treatment: F_(24, 120)_ = 6.51, *p* < 0.05; Figure 2A,B) (multiple comparison test, P1: F_(2, 15)_ = 15.62, *p* < 0.05; P2: F_(2, 15)_ = 14.14, *p* < 0.05; P3: F_(2, 15)_ = 13.87, *p* < 0.05; Table 2A), rearing activity (two-way RM-ANOVA, Time: F_(12, 60)_ = 28.47, *p* < 0.05; Treatment: F_(2, 10)_ = 22.24, *p* < 0.05; Time × Treatment: F_(24, 120)_ = 6.23, *p* < 0.05; Figure 2D) (P1: F_(2, 15)_ = 13.57, *p* < 0.05; P2: F_(2, 15)_ = 18.42, *p* < 0.05; P3: F_(2, 15)_ = 19.06, *p* < 0.05; Table 2B), and rotational activity (two-way RM-ANOVA, Time: F_(12, 60)_ = 29.92, *p* < 0.05; Treatment: F_(2, 10)_ = 34.03, *p* < 0.05; Time × Treatment: F_(24, 120)_ = 5.14, *p* < 0.05; Figure 2F) (multiple comparison test, P1: F_(2, 15)_ = 15.62, *p* < 0.05; P2: F_(2, 15)_ = 19.12, *p* < 0.05; P3: F_(2, 15)_ = 21.61, *p* < 0.05; Table 2C) compared to the vehicle and nicotine control groups.

Similar to KCSC A, repeated KCSC B administration also induced prolonged increases in ambulatory activity (two-way RM-ANOVA, Time: F_(12, 60)_ = 46.29, *p* < 0.05; Treatment: F(2, 10) = 41.15, *p* < 0.05; Time × Treatment: F_(24, 120)_ = 7.59, *p* < 0.05; Figure 2A,C) (multiple comparison test, P1: F_(2, 15)_ = 21.74, *p* < 0.05; P2: F_(2, 15)_ = 16.83, *p* < 0.05; P3: F_(2, 15)_ = 15.56, *p* < 0.05; Table 2A), rearing activity (two-way RM-ANOVA, Time: F_(12, 60)_ = 22.74, *p* < 0.05; Treatment: F_(2, 10)_ = 30.22, *p* < 0.05; Time × Treatment: F_(24, 120)_ = 4.30, *p* < 0.05; Figure 2E) (multiple comparison test, P1: F_(2, 15)_ = 10.09, *p* < 0.05; P2: F_(2, 15)_ = 14.24, *p* < 0.05; P3: F_(2, 15)_ = 22.25, *p* < 0.05; Table 2B), and rotational activity (two-way RM-ANOVA, Time: F_(12, 60)_ = 40.34, *p* < 0.05; Treatment: F_(2, 10)_ = 47.64, *p* < 0.05; Time × Treatment: F_(24, 120)_ = 6.65, *p* < 0.05; Figure 2G) (multiple comparison test, P1: F_(2, 15)_ = 10.09, *p* < 0.05; P2: F_(2, 15)_ = 14.24, *p* < 0.05; P3: F_(2, 15)_ = 22.25, *p* < 0.05; Table 2C) compared to the vehicle and nicotine control groups. Accumulative changes in ambulatory, rearing, and rotational activities at P1, P2, and P3 after 14 days of repeated administration of vehicle, nicotine, KCSC A, and KCSC B are listed in Table 2.

### 3.4. Repeated Administration of KCSCs Induced Prolonged Increases in Ambulatory and Stereotypy Times, and Prolonged Decrease in Resting Time in Behavioral States

The results showed that repeated administration of KCSC A produced a prolonged increase in ambulatory time (two-way RM-ANOVA, Time: F_(12, 60)_ = 52.94, *p* < 0.05; Treatment: F_(2, 10)_ = 32.79 *p* < 0.05; Time × Treatment: F_(24, 120)_ = 5.47, *p* < 0.05; Figure 2H) (multiple comparison test, P1: F_(2, 15)_ = 39.85, *p* < 0.05; P2: F_(2, 15)_ = 21.23, *p* < 0.05; P3: F_(2, 15)_ = 25.97, *p* < 0.05; Supplementary Table S1A) and a prolonged decrease in resting time (two-way RM-ANOVA, Time: F_(12, 60)_ = 34.20, *p* < 0.05; Treatment: F_(2, 10)_ = 29.27, *p* < 0.05; Time × Treatment: F_(24, 120)_ = 4.45, *p* < 0.05; Figure 2L) (multiple comparison test; P1: F_(2, 15)_ = 31.15, *p* < 0.05; P2: F_(2, 15)_ = 21.91, *p* < 0.05; P3: F_(2, 15)_ = 18.93, *p* < 0.05; Supplementary Table S1C) compared to the vehicle and nicotine control groups. Additionally, repeated administration of KCSC A produced a tendency to increase stereotypy time (two-way RM-ANOVA, Time: F_(12, 60)_ = 6.52, *p* < 0.05; Treatment: F_(2, 10)_ = 83.37, *p* < 0.05; Time × Treatment: F_(24, 120)_ = 4.17, *p* < 0.05; Figure 2J) compared to nicotine control group. However, it was only significant at the 60 min time-point (two-way RM-ANOVA, Time: F_(12, 60)_ = 6.52, *p* < 0.05; Treatment: F_(2, 10)_ = 83.37, *p* < 0.05; Time × Treatment: F_(24, 120)_ = 4.17, *p* < 0.05; Figure 2J) (multiple comparison test; P1: F_(2, 15)_ = 20.88, *p* < 0.05; P2: F_(2, 15)_ = 28.89, *p* < 0.05; P3: F_(2, 15)_ = 18.86, *p* < 0.05; Supplementary Table S1B).

In the KCSC B group, repeated administration of KCSC B induced a prolonged decrease in resting time (two-way RM-ANOVA, Time: F_(12, 60)_ = 36.53, *p* < 0.05; Treatment: F_(2, 10)_ = 81.96, *p* < 0.05; Time × Treatment: F_(24, 120)_ = 4.83, *p* < 0.05; Figure 2M) (P1: F_(2, 15)_ = 79.02, *p* < 0.05; P2: F_(2, 15)_ = 25.36, *p* < 0.05; P3: F_(2, 15)_ = 21.21, *p* < 0.05; Supplementary Table S1C) compared to the vehicle and nicotine control groups. However, the results showed that repeated administration of KCSC B tended to increase ambulatory time (two-way RM-ANOVA, Time: F_(12, 60)_ = 56.98, *p* < 0.05; Treatment: F(2, 10) = 73.94, *p* < 0.05; Time × Treatment: F_(24, 120)_ = 6.77, *p* < 0.05; Figure 2I) (multiple comparison test, P1: F_(2, 15)_ = 52.47, *p* < 0.05; P2: F_(2, 15)_ = 22.56, *p* < 0.05; P3: F_(2, 15)_ = 25.78, *p* < 0.05; Supplementary Table S1A) and stereotypy time (two-way RM-ANOVA, Time: F_(12, 60)_ = 6.32, *p* < 0.05; Treatment: F_(2, 10)_ = 83.85, *p* < 0.05; Time × Treatment: F_(24, 120)_ = 4.21, *p* < 0.05; Figure 2K) (multiple comparison test, P1: F_(2, 15)_ = 28.90, *p* < 0.05; P2: F_(2, 15)_ = 21.97, *p* < 0.05; P3: F_(2, 15)_ = 18.35, *p* < 0.05; Supplementary Table S1B) compared to nicotine, but it was also only significant at 60 min and 55 min time-points. Accumulative changes in ambulatory, stereotypy, and resting time at P1, P2, and P3 after 14 days of repeated administration of vehicle, nicotine, KCSC A, and KCSC B are listed in Supplementary Table S1.

### 3.5. Challenge Administration of KCSCs after Withdrawal Periods Increased Ambulatory, Rearing, and Rotational Activities

**Figure 3:**
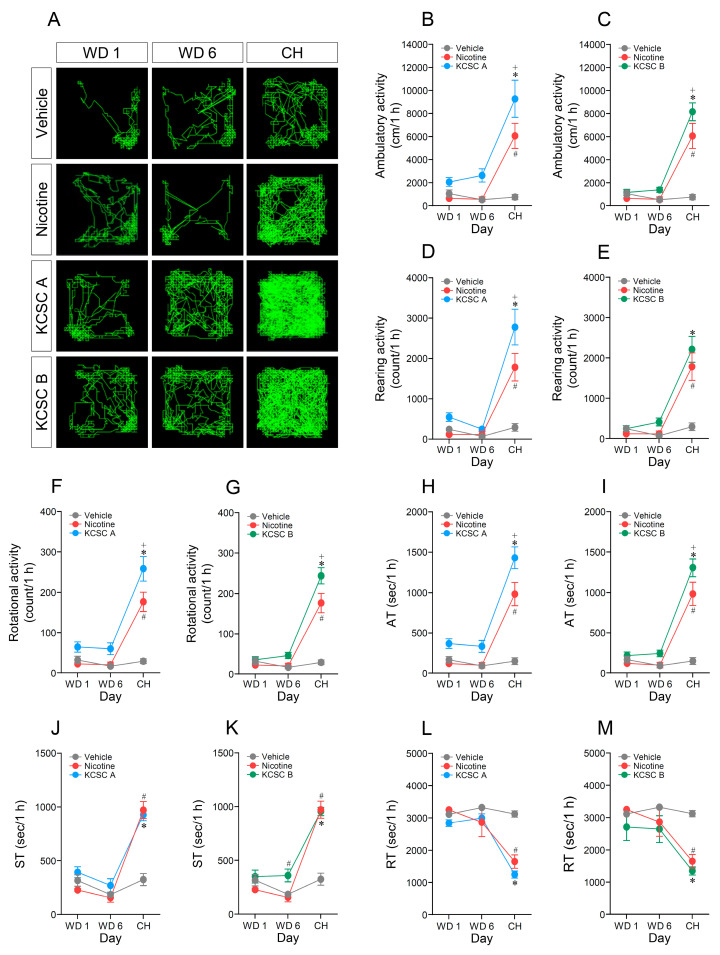
Changes in behavioral activities after withdrawal or challenge administration of two KCSCs. Statistical significance was determined by two-way RM-ANOVA and multiple comparison test with Bonferroni’s post-hoc test. ^#^ *p* < 0.05, vehicle challenge group vs. nicotine challenge group; * *p* < 0.05, vehicle challenge group vs. KCSC challenge groups; ^+^ *p* < 0.05, nicotine challenge group vs. KCSC challenge groups. WD, withdrawal; CH, challenge; AT, ambulatory time; ST, stereotypy time; RT, resting time. *n* = 6 per group.

Since re-exposure to nicotine after nicotine abstinence increased psychomotor behaviors [8], open-field tests whether challenge administration of KCSCs after drug abstinence period induces psychomotor sensitization on ambulatory, rearing, and rotational activities were performed. The results showed that challenge administration of KCSC A increased ambulatory activity (two-way RM-ANOVA, Time: F_(2, 45)_ = 34.32, *p* < 0.05; Treatment: F_(2, 45)_ = 23.10, *p* < 0.05; Time × Treatment: F_(4, 45)_ = 9.25, *p* < 0.05) (multiple comparison test, challenge: F_(2, 15)_ = 14.62, *p* < 0.05) (Figure 3A,B) compared to the vehicle and nicotine control groups. Similarly, challenge administration of KCSC B also increased ambulatory activity (two-way RM-ANOVA, Time: F_(2, 45)_ = 72.18, *p* < 0.05; Treatment: F_(2, 45)_ = 25.28, *p* < 0.05; Time × Treatment: F_(4, 45)_ = 19.24, *p* < 0.05) (multiple comparison test, challenge: F_(2, 15)_ = 24.01, *p* < 0.05) (Figure 3A,C) compared to the vehicle and nicotine control groups. KCSC A group showed a tendency to increase ambulatory activity at withdrawal day 6 compared to the nicotine group but there was no significant difference (Figure 3A,B).

Challenge administration of KCSC A increased rearing activity (two-way RM-ANOVA, Time: F_(2, 45)_ = 51.06, *p* < 0.05; Treatment: F_(2, 45)_ = 19.12, *p* < 0.05; Time × Treatment: F_(4, 45)_ = 11.54, *p* < 0.05) (multiple comparison test, challenge: F_(2, 15)_ = 14.62, *p* < 0.05) (Figure 3D) compared to the vehicle and nicotine control groups. However, there was no difference in the rearing activity between nicotine and KCSC B challenge administration groups (two-way RM-ANOVA, Time: F_(2, 45)_ = 53.72, *p* < 0.05; Treatment: F_(2, 45)_ = 15.48, *p* < 0.05; Time × Treatment: F_(4, 45)_ = 11.02, *p* < 0.05) (multiple comparison test, challenge: F_(2, 15)_ = 13.43, *p* < 0.05) (Figure 3E). Similar to the ambulatory activity, challenge administration of KCSC A (two-way RM-ANOVA, Time: F_(2, 45)_ = 61.54, *p* < 0.05; Treatment: F_(2, 45)_ = 33.67, *p* < 0.05; Time × Treatment: F_(4, 45)_ = 14.83, *p* < 0.05) (multiple comparison test, challenge: F_(2, 15)_ = 26.18, *p* < 0.05) (Figure 3F) and KCSC B increased the rotational activity (two-way RM-ANOVA, Time: F_(2, 45)_ = 103.20, *p* < 0.05; Treatment: F_(2, 45)_ = 36.08, *p* < 0.05; Time × Treatment: F_(4, 45)_ = 25.49, *p* < 0.05) (multiple comparison test, challenge: F_(2, 15)_ = 35.91, *p* < 0.05) (Figure 3G) compared to the vehicle and nicotine control groups. However, there was no difference in rearing and rotational activities among the groups during withdrawal periods (Figure 3D–G).

### 3.6. Challenge Administration of KCSCs also Increased Ambulatory and Stereotypy Time, but Decreased Resting Time in Behavioral States

The results revealed that challenge administration of KCSC A increased ambulatory time (two-way RM-ANOVA, Time: F_(2, 45)_ = 73.51, *p* < 0.05; Treatment: F_(2, 45)_ = 41.89, *p* < 0.05; Time × Treatment: F_(4, 45)_ = 17.84, *p* < 0.05) (multiple comparison test, challenge: F_(2, 15)_ = 30.76, *p* < 0.05) (Figure 3H) compared to the vehicle and nicotine control groups. Similarly, challenge administration of KCSC B also increased ambulatory time (two-way RM-ANOVA, Time: F_(2, 45)_ = 93.52, *p* < 0.05; Treatment: F_(2, 45)_ = 33.52, *p* < 0.05; Time × Treatment: F_(4, 45)_ = 22.82, *p* < 0.05) (multiple comparison test, challenge: F_(2, 15)_ = 30.67, *p* < 0.05) (Figure 3I) compared to vehicle and nicotine control groups. Time spent in stereotypy behaviors was also increased after challenge administration of KCSC A (two-way RM-ANOVA, Time: F_(2, 45)_ = 91.07, *p* < 0.05; Treatment: F_(2, 45)_ = 19.22, *p* < 0.05; Time × Treatment: F_(4, 45)_ = 16.88, *p* < 0.05) (multiple comparison test, challenge: F_(2, 15)_ = 31.39, *p* < 0.05) and KCSC B (two-way RM-ANOVA, Time: F_(2, 45)_ = 92.23, *p* < 0.05; Treatment: F_(2, 45)_ = 23.21, *p* < 0.05; Time × Treatment: F_(4, 45)_ = 17.86, *p* < 0.05) (multiple comparison test, challenge: F_(2, 15)_ = 36.91, *p* < 0.05) compared to the vehicle control group (Figure 3J,K). Interestingly, KCSC B increased stereotypy time at withdrawal day 6 (withdrawal day 6: F_(2, 15)_ = 6.52, *p* < 0.05) compared to the vehicle and nicotine control groups (Figure 3K). In contrast, time spent in resting behaviors was decreased after challenge administration of KCSC A (two-way RM-ANOVA, Time: F_(2, 45)_ = 34.01, *p* < 0.05; Treatment: F_(2, 45)_ = 16.61, *p* < 0.05; Time × Treatment: F_(4, 45)_ = 8.05, *p* < 0.05) (multiple comparison test, challenge: F_(2, 15)_ = 45.71, *p* < 0.05) and KCSC B (two-way RM-ANOVA, Time: F_(2, 45)_ = 13.05, *p* < 0.05; Treatment: F_(2, 45)_ = 10.18, *p* < 0.05; Time × Treatment: F_(4, 45)_ = 2.96, *p* < 0.05) (multiple comparison test, challenge: F_(2, 15)_ = 39.75, *p* < 0.05) compared to the vehicle control group (Figure 3L,M). Relative ratios of ambulatory, stereotypy, and resting times (ambulatory time: stereotypy time: resting time) in the behaviors after challenge administration of vehicle, nicotine, KCSC A, and KCSC B were as following: (1) Vehicle (4.17:9.02:86.81); (2) nicotine (27.29:27.00:45.71); (3) KCSC A (39.71:25.76:34.53); (4) KCSC B (36.24:26.54:37.22) (Supplementary Figure S4).

### 3.7. Challenge Administration of KCSCs Produced Prolonged Increases in Ambulatory, Rearing, and Rotational Activities

**Figure 4:**
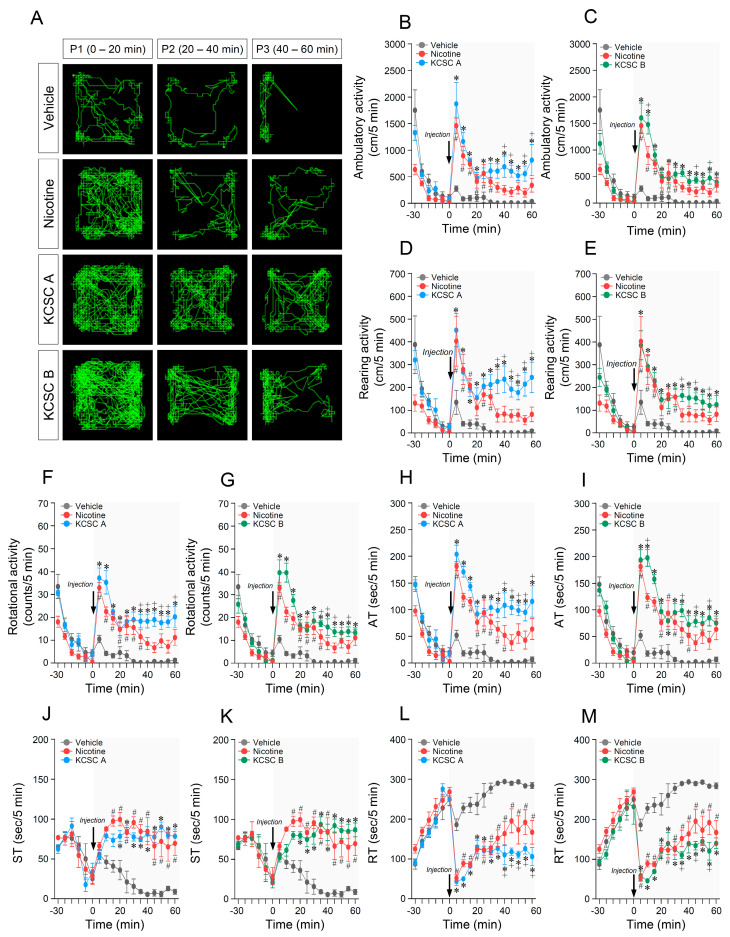
Prolonged changes in behavioral activities after challenge administration of two KCSCs. Statistical significance was determined by two-way RM-ANOVA and multiple comparison test with Bonferroni’s post-hoc test. ^#^ *p* < 0.05, vehicle challenge group vs. nicotine challenge group; * *p* < 0.05, vehicle challenge group vs. KCSC challenge groups; ^+^ *p* < 0.05, nicotine challenge group vs. KCSC challenge groups. WD, withdrawal; CH, challenge; AT, ambulatory time; ST, stereotypy time; RT, resting time. *n* = 6 per group.

The results showed that challenge administration of KCSC A produced prolonged increase in ambulatory activity (two-way RM-ANOVA, Time: F_(12, 60)_ = 27.58, *p* < 0.05; Treatment: F_(2, 10)_ = 13.66, *p* < 0.05; Time × Treatment: F_(24, 120)_ = 5.04, *p* < 0.05; Figure 4A,B) (multiple comparison test, P1: F_(2, 15)_ = 18.49, *p* < 0.05; P2: F_(2, 15)_ = 9.09, *p* < 0.05; P3: F_(2, 15)_ = 11.69, *p* < 0.05; Table 3A), rearing activity (two-way RM-ANOVA, Time: F_(12, 60)_ = 16.23, *p* < 0.05; Treatment: F_(2, 10)_ = 13.10, *p* < 0.05; Time × Treatment: F_(24, 120)_ = 3.00, *p* < 0.05; Figure 4D) (multiple comparison test, P1: F_(2, 15)_ = 10.05, *p* < 0.05; P2: F_(2, 15)_ = 15.33, *p* < 0.05; P3: F_(2, 15)_ = 16.08, *p* < 0.05; Table 3B), and rotational activity (two-way RM-ANOVA, Time: F_(12, 60)_ = 30.56, *p* < 0.05; Treatment: F_(2, 10)_ = 24.72, *p* < 0.05; Time × Treatment: F_(24, 120)_ = 5.14, *p* < 0.05; Figure 4F) (multiple comparison test, P1: F_(2, 15)_ = 35.22, *p* < 0.05; P2: F_(2, 15)_ = 17.19, *p* < 0.05; P3: F_(2, 15)_ = 17.64, *p* < 0.05; Table 3C) compared to the vehicle and nicotine control groups.

In the KCSC B group, challenge administration of KCSC B also induced prolonged increases in ambulatory activity (two-way RM-ANOVA, Time: F_(12, 60)_ = 27.91, *p* < 0.05; Treatment: F_(2, 10)_ = 27.47, *p* < 0.05; Time × Treatment: F_(24, 120)_ = 7.77, *p* < 0.05; Figure 4A,C) (multiple comparison test, P1: F_(2, 15)_ = 29.08, *p* < 0.05; P2: F_(2, 15)_ = 9.38, *p* < 0.05; P3: F_(2, 15)_ = 11.57, *p* < 0.05, Table 3A), rearing activity (two-way RM-ANOVA, Time: F_(12, 60)_ = 20.62, *p* < 0.05; Treatment: F_(2, 10)_ = 12.96, *p* < 0.05; Time × Treatment: F_(24, 120)_ = 2.36, *p* < 0.05; Figure 4E) (multiple comparison test, P1: F_(2, 15)_ = 9.25, *p* < 0.05; P2: F_(2, 15)_ = 10.02, *p* < 0.05; P3: F_(2, 15)_ = 12.74, *p* < 0.05; Table 3B), and rotational activity (two-way RM-ANOVA, Time: F_(12, 60)_ = 25.22, *p* < 0.05; Treatment: F_(2, 10)_ = 38.82, *p* < 0.05; Time × Treatment: F_(24, 120)_ = 7.08, *p* < 0.05; Figure 4G) (multiple comparison test, P1: F_(2, 15)_ = 59.63, *p* < 0.05; P2: F_(2, 15)_ = 13.78, *p* < 0.05; P3: F_(2, 15)_ = 15.16, *p* < 0.05; Table 3C) compared to the vehicle and nicotine control groups. Accumulative changes in ambulatory, rearing, and rotational activities at P1, P2, and P3 periods after challenge administration of vehicle, nicotine, KCSC A, and KCSC B are listed in Table 3.

### 3.8. Challenge Administration of KCSCs Induced Prolonged Increases in Ambulatory and Stereotypy Times, and a Prolonged Decrease in Resting Time in Behavioral States

The results showed that challenge administration of KCSC A produced a prolonged increase in ambulatory time (two-way RM-ANOVA, Time: F_(12, 60)_ = 35.77, *p* < 0.05; Treatment: F_(2, 10)_ = 25.75, *p* < 0.05; Time × Treatment: F_(24, 120)_ = 5.08, *p* < 0.05; Figure 4H) (multiple comparison test, P1: F_(2, 15)_ = 77.57, *p* < 0.05; P2: F_(2, 15)_ = 15.11, *p* < 0.05; P3: F_(2, 15)_ = 15.97, *p* < 0.05; Supplementary Table S2A) and a prolonged decrease in resting time (two-way RM-ANOVA, Time: F_(12, 60)_ = 25.97, *p* < 0.05; Treatment: F_(2, 10)_ = 36.82, *p* < 0.05; Time × Treatment: F_(24, 120)_ = 4.54, *p* < 0.05; Figure 4L) (multiple comparison test, P1: F_(2, 15)_ = 57.74, *p* < 0.05; P2: F_(2, 15)_ = 32.66, *p* < 0.05; P3: F_(2, 15)_ = 23.45, *p* < 0.05; Supplementary Table S2C) compared to the vehicle and nicotine control groups. However, there was no significant difference in stereotypy time between challenge administration of nicotine and KCSC B groups (Figure 4J and Supplementary Table S2B).

Similarly, challenge administration of KCSC B also induced a prolonged increase in ambulatory time (two-way RM-ANOVA, Time: F_(12, 60)_ = 37.52, *p* < 0.05; Treatment: F_(2, 10)_ = 32.48, *p* < 0.05; Time × Treatment: F_(24, 120)_ = 6.70, *p* < 0.05; Figure 4I) (multiple comparison test, P1: F_(2, 15)_ = 77.73, *p* < 0.05; P2: F_(2, 15)_ = 10.84, *p* < 0.05; P3: F_(2, 15)_ = 13.16, *p* < 0.05; Supplementary Table S2A) compared to the vehicle and nicotine control groups. However, challenge administration of KCSC B induced a prolonged increase in stereotypy time (two-way RM-ANOVA, Time: F_(12, 60)_ = 4.15, *p* < 0.05; Treatment: F_(2, 10)_ = 28.11, *p* < 0.05; Time × Treatment: F_(24, 120)_ = 6.29, *p* < 0.05; Figure 4K) (multiple comparison test, P1: F_(2, 15)_ = 5.91, *p* < 0.05; P2: F_(2, 15)_ = 44.88, *p* < 0.05; P3: F_(2, 15)_ = 15.13, *p* < 0.05; Supplementary Table S2B) and a prolonged decrease in resting time (two-way RM-ANOVA, Time: F_(12, 60)_ = 17.13, *p* < 0.05; Treatment: F_(2, 10)_ = 33.50, *p* < 0.05; Time × Treatment: F_(24, 120)_ = 3.33, *p* < 0.05; Figure 4M) (multiple comparison test, P1: F_(2, 15)_ = 63.17, *p* < 0.05; P2: F_(2, 15)_ = 23.62, *p* < 0.05; P3: F_(2, 15)_ = 18.81, *p* < 0.05; Supplementary Table S2C) compared to the vehicle control group, but not nicotine group. Accumulative changes in ambulatory, stereotypy, and resting time at P1, P2, and P3 after challenge administration of vehicle, nicotine, KCSC A, and KCSC B are listed in Supplementary Table S2.

### 3.9. In Vitro Calibration of Real-Time Glutamate Biosensing

Glutamate biosensors were calibrated and control experiments were conducted before and after real-time glutamate biosensing. The mean of sensitivity of glutamate biosensors before measurements was 0.711 ± 0.045 nA/μM, and an approximate two-fold decrease (0.424 ± 0.043 nA/μM) in glutamate sensitivity was observed after measurements (Supplementary Figure S5A). Linear calibration plots were obtained using steady-state currents and glutamate concentrations from 0 to 4 μM (Supplementary Figure S5B). In in vitro calibration, there were no changes in currents and glutamate concentrations in response to the addition of glutamate standard solutions within the glutamate null sensors (before biosensing 0.021 ± 0.004 nA/μM; after biosensing 0.009 ± 0.001 nA/μM) (Supplementary Figure S5B).

### 3.10. Repeated Administration of KCSCs Increased Glutamate Concentration in the Dorsal Striatum

**Figure 5:**
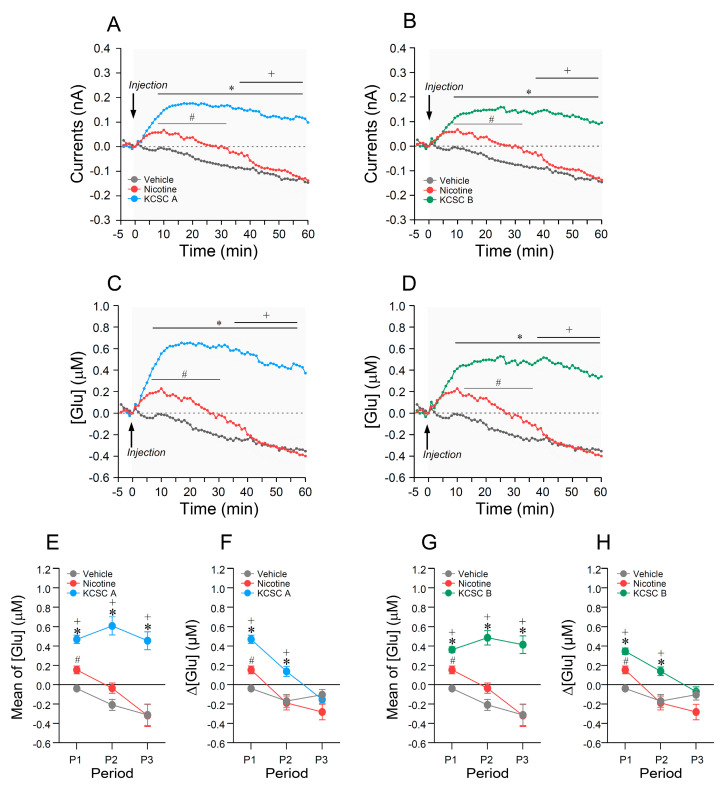
Changes in glutamate responses in the dorsal striatum after the repeated administration of two KCSCs using real-time glutamate biosensing. Statistical significance was determined by two-way RM-ANOVA and multiple comparison test with Bonferroni’s post-hoc test. ^#^ *p* < 0.05, repeated vehicle group vs. repeated nicotine group; * *p* < 0.05, repeated vehicle group vs. repeated KCSC groups; ^+^ *p* < 0.05, repeated nicotine group vs. repeated KCSC groups. *n* = 6 per group.

Since repeated administration of nicotine or 3R4F CSC induced behavioral sensitization via increased glutamate response in the dorsal striatum [8,29], extracellular glutamate concentrations in the dorsal striatum was measured to investigate the effect of glutamate response on psychomotor sensitization after repeated administration of KCSCs. The results showed that repeated administration of KCSC A increased currents (two-way RM-ANOVA, Time: F_(60, 300)_ = 13.60, *p* < 0.05; Treatment: F_(2, 10)_ = 36.92, *p* < 0.05; Time × Treatment: F_(120, 600)_ = 6.65, *p* < 0.05) (Figure 5A) and glutamate concentrations ([Glu]) (two-way RM-ANOVA, Time: F_(60, 300)_ = 9.91, *p* < 0.05; Treatment: F_(2, 10)_ = 39.91, *p* < 0.05; Time × Treatment: F_(120, 600)_ = 7.73, *p* < 0.05) (Figure 5C) compared to the vehicle and nicotine control groups. In additional analysis, changes in [Glu] was divided into three different time periods (P1: 0–20 min, P2: 20–40 min, P3: 40–60 min), repeated administration of KCSC A increased mean [Glu] at P1, P2, and P3 (multiple comparison test, P1: F_(2, 15)_ = 46.10, *p* < 0.05; P2: F_(2, 15)_ = 38.20, *p* < 0.05; P3: F_(2, 15)_ = 17.55, *p* < 0.05) (Figure 5E). Rates of [Glu] change after repeated KCSC A administration were also increased at P1 and P2 (multiple comparison test, P1: F_(2, 15)_ = 46.10, *p* < 0.05; P2: F_(2, 15)_ = 8.18, *p* < 0.05), but not at P3 compared to the vehicle and nicotine control groups (Figure 5F).

Similarly, repeated administration of KCSC B also increased currents (two-way RM-ANOVA, Time: F_(60, 300)_ = 10.45, *p* < 0.05; Treatment: F_(2, 10)_ = 33.99, *p* < 0.05; Time × Treatment: F_(120, 600)_ = 7.03, *p* < 0.05) (Figure 5B), and [Glu] (two-way RM-ANOVA, Time: F_(60, 300)_ = 7.99, *p* < 0.05; Treatment: F_(2, 10)_ = 39.24, *p* < 0.05; Time × Treatment: F_(120, 600)_ = 7.58, *p* < 0.05) (Figure 5D) compared to the vehicle and nicotine control groups. Repeated administration of KCSC B also increased mean [Glu] at P1, P2, and P3 periods (multiple comparison test, P1: F_(2, 15)_ = 31.44, *p* < 0.05; P2: F_(2, 15)_ = 34.20, *p* < 0.05; P3: F_(2, 15)_ = 15.81, *p* < 0.05) (Figure 5G), and rates of [Glu] change at P1 and P2 (multiple comparison test, P1: F_(2, 15)_ = 31.44, *p* < 0.05; P2: F_(2, 15)_ = 8.69, *p* < 0.05), but not at P3 (Figure 5H) compared to the vehicle and nicotine control groups. Absolute [Glu] means and rates of [Glu] change at P1, P2, and P3 periods after 14 days of repeated administration of KCSC A and KCSC B are listed in Table 4.

### 3.11. Relations between Rates of Change in [Glu] in the Dorsal Striatum and Psychomotor Sensitization Following Repeated Administration of KCSCs

**Figure 6:**
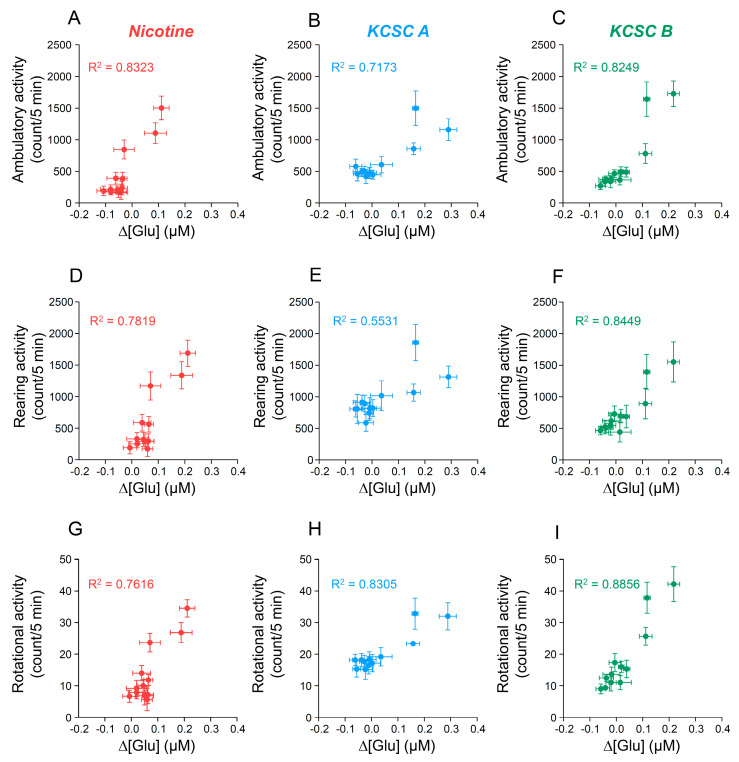
Correlation coefficients for the relationships between the rate of change in glutamate concentration (Δ[Glu]) of the dorsal striatum and psychomotor behaviors after 14 days of repeated administration of two KCSCs.

To investigate the relationship between the repeated KCSC-induced increases in glutamate response in the dorsal striatum and psychomotor sensitization, we performed Pearson’s correlation analysis [8,29,41]. The repeated nicotine group showed strong correlations between rate of [Glu] change and ambulatory activity (R^2^ = 0.8323, *p* = 0.001) (Figure 6A), rearing activity (R^2^ = 0.7819, *p* = 0.001) (Figure 6D), and rotational activity (R^2^ = 0.7616, *p* = 0.002) (Figure 6G). Similarly, the rates of [Glu] change after repeated administration of KCSC A were strongly correlated with ambulatory activity (R^2^ = 0.7173, *p* = 0.001) (Figure 6B), rearing activity (R^2^ = 0.5531, *p* = 0.006) (Figure 6E), and rotational activity (R^2^ = 0.8305, *p* = 0.001) (Figure 6H). In the repeated KCSC B group, the result showed that rates of [Glu] change were strongly correlated with ambulatory activity (R^2^ = 0.8249, *p* = 0.001) (Figure 6C), rearing activity (R^2^ = 0.8449, *p* = 0.001) (Figure 6F), and rotational activity (R^2^ = 0.8856, *p* = 0.001) (Figure 6I).

## 4. Discussion

Tobacco plants and products, such as cigarettes and chewing tobacco, contain a psychoactive compound, nicotine as well as non-nicotine alkaloids, such as nornicotine, cotinine, anabasine, anatabine, and etc. [10,11,12,13]. Previous studies demonstrated that the content and ratio of nicotine and non-nicotine alkaloids in cigarette products differ significantly from brand to brand, but in general, most cigarette products consist of high amount of nicotine (more than 90%) and also contain non-nicotine alkaloids including nornicoitne, anatabine, and anabasine [12,13]. Consistently, results from this study demonstrated that KCSC A and KCSC B included more than 95% of nicotine as well as non-nicotine alkaloids, such as nornicotine (KCSC A: 0.49%, KCSC B: 0.47%), cotinine (KCSC A: 0.12%, KCSC B: 0.15%), anatabine (KCSC A: 3.40%, KCSC B: 3.46%), anabasine (KCSC A: 0.10%, KCSC B: 0.08%), myosmine (KCSC A: 0.02%, KCSC B: 0.02%), norharmane (KCSC A: 0.01%, KCSC B: 0.01%), and harmane (KCSC A: 0.01%, KCSC B: 0.01%). Taken together, these findings suggest that the two different commercial cigarette products in South Korea also contained psychoactive components, such as nicotine and non-nicotine alkaloids like other cigarette products.

It is well-known that non-nicotine tobacco constituents act on nAChRs in the reward system as well as nicotine due to their structural similarities to nicotine, resulting in inducing nicotine-like behavioral effects in rodents [14,15,17]. Previous studies demonstrated that each of these non-nicotine alkaloids differentially regulates reinforcing properties and discriminative-stimulus effects of nicotine in self-administration and drug-discrimination paradigms, respectively [17,18,19]. Furthermore, exposure to cigarette smoke enhances discriminative-stimulus effects more than exposure to nicotine alone [42]. Our previous study also demonstrated that repeated exposure to cigarette smoke produced hypersensitization of psychomotor behaviors more than nicotine alone, and these increases in psychomotor activities were more prolonged than nicotine alone [29]. Consistent with these findings, we observed that repeated exposure to KCSC A and KCSC B significantly increased ambulatory, rearing, and rotational activities compared to repeated nicotine alone, and these increases in psychomotor activities were significantly sustained compared to nicotine alone. These findings suggest that cigarette smoke of commercial cigarette brands also has stronger psychoactive properties on psychomotor behaviors compared to those of nicotine alone. Furthermore, repeated exposure to KCSC A and KCSC B also increased time spent in ambulatory and stereotypy behaviors compared to repeated exposure to nicotine alone. Based on these findings, it is possible to consider the stronger psychoactive effects of cigarette smoke on psychomotor behaviors are due to long-lasting synergistic actions of nicotine and non-nicotine alkaloids on nAChRs in the dorsal striatum.

Re-exposure to drugs after drug abstinence is one of the major factors causing the recurrence of psychoactive behavioral changes, such as behavioral sensitization through drug-induced physiological changes in the reward system [26,43]. In our previous study, re-exposure to nicotine after nicotine withdrawal period induces a relapse of psychomotor sensitization, which is similar degree of sensitized behaviors by repeated nicotine exposure [8]. Consistently, our results demonstrated that challenge administration of KCSC A and KCSC B produced psychomotor sensitization in ambulatory, rearing, and rotational activities and time spent in ambulatory and stereotypy behaviors similar to those of repeated KCSC A and KCSC B administration. Additionally, the increases in psychomotor behaviors by challenge administration of KCSC A and KCSC B were higher than challenge nicotine administration. Taken together, these findings suggest that re-exposure to cigarette smoke is crucial to reinstate psychomotor sensitization and has a higher relapse potential to tobacco dependence compared to re-exposure to nicotine alone. The present data show that administration of KCSC A produced a slight increase in ambulatory activity, while administration of KCSC B produced a significant increase in stereotypy time at the sixth withdrawal day compared to the vehicle and nicotine control groups, suggesting that different pharmacokinetics of the two KCSCs in psychomotor sensitization, even though we do not have any supporting evidence.

It is well-known that glutamatergic neurotransmission is involved in the development of drug dependence through the mediation of synaptic plasticity [44,45]. The dorsal striatum is a forebrain structure that integrates nigrostriatal dopaminergic and corticostriatal glutamatergic neurotransmissions and is crucial in drug-mediated dependence, motivated behavior, and habitual behaviors [8,30,46,47]. Exposure to psychoactive drugs, such as nicotine or cocaine, increases glutamatergic response in the dorsal striatum and increases addictive behaviors including behavioral sensitization [8,30,48,49,50]. In a previous study, repeated administration of CSC extracted from 3R4F Kentucky reference cigarette caused more prolonged increase in glutamate concentration in the dorsal striatum compared to nicotine alone [29]. Similarly, in this study, it was found that repeated exposure to KCSC A and KCSC B increased extracellular glutamate concentrations in the dorsal striatum more than nicotine alone. Considering that both of KCSC A and KCSC B contained non-nicotine alkaloids as well as nicotine, it is possible to conclude that the prolonged potentiation of glutamate response in the dorsal striatum by repeated exposure to cigarette smoke was due to hyperstimulation of nAChRs by synergistic actions of nicotine and non-nicotine alkaloids in cigarette smoke. However, there is a possibility that potentiation of dopaminergic response contributes to elevate glutamatergic response via trans-synaptic activation of forebrain basal ganglia [44]. Additionally, the glutamate response in other brain regions, such as the NAc, amygdala, and hippocampus is also closely related to the development of drug-dependent behaviors [24,51,52]. For this reason, the relationship between changes in glutamate release in these brain regions and cigarette smoke-induced hypersensitization of behavior should be determined in further studies.

Drug-induced psychomotor sensitization has been shown to be related to hyperactivation of glutamatergic neurotransmission in the dorsal striatum and NAc [8,29,53]. Consistent with these findings, the present study showed that the increases in psychomotor behaviors (ambulatory, rearing, and rotational activities) were highly correlated with changes in glutamate concentrations in the dorsal striatum following the repeated exposure to KCSC A and KCSC B. Taken together, these findings suggest that the long-lasting glutamatergic response in the dorsal striatum may contribute to prolonged hypersensitization of psychomotor behaviors following exposure to commercial cigarette smoke.

Individual administration of each non-nicotine alkaloid, such as cotinine, myosmine, and anatabine produced distinctive patterns of effects on ambulatory activity that were time and dose-dependent [54]. Additionally, treatment of anatabine, cotinine, or myosmine increases nicotine-induced general activity [18]. In self-administration paradigm, rats receiving nicotine with minor alkaloids produced a higher number of reinforcing effects and hypersensitization of ambulatory activity than those of nicotine alone [18]. Taken together, these findings suggest that the type of the compound exposed with the nicotine and the amount of nicotine exposed are important to alter the psychoactive properties of nicotine. Consistently, the present results showed that the relative ratio of nicotine and non-nicotine alkaloids in the KCSC A and KCSC B was slightly different. Additionally, the changes in hyperactivation of glutamate response and hypersensitization of psychomotor behaviors (frequency and duration) by exposure to KCSC A and KCSC B showed a similar pattern, but slightly different in degree. Taken together, these findings suggest that the onset of tobacco-associated glutamate response and behavioral changes depends on the levels and composition ratio of psychoactive compounds contained in commercial cigarette products.

## 5. Conclusions

Repeated and challenge administration of KCSCs prepared from two commercial cigarettes caused more sustained increases in psychomotor behaviors and ambulatory times than the repeated and challenge administration of nicotine alone. In real-time glutamate biosensing, the repeated administration of KCSCs induced prolonged increases in extracellular glutamate concentration in the dorsal striatum. These prolonged increases in psychomotor behaviors and glutamate response by repeated administration of KCSCs are strongly and positively correlated. Taken together, these data suggest that the prolonged increases in glutamate response of the dorsal striatum and ambulatory times in behavioral states result in hypersensitization of psychomotor behaviors, which are induced by the synergistic effects of nicotine and non-nicotine alkaloids in commercial cigarette smoke. In addition, the changes in glutamate response in the dorsal striatum by repeated exposure to commercial cigarettes may contribute to neuroadaptation during withdrawal period, which is closely related to behavioral sensitization in relapse. In-depth approaches in exploring the glutamate receptor-mediated neurotransmission in psychomotor sensitization after repeated and challenge exposure to commercial cigarette smoke or withdrawal are needed in further study.

## Figures and Tables

**Table 1:** Nicotine and non-nicotine alkaloid contents of the two commercial cigarette brands in South Korea (KCSCs) (μg/mL).

Types	Nicotine	Nornicotine	Cotinine	Anabasine	Anatabine	Myosmine	Norharmane	Harmane
KCSC A	5640.0 (95.85)	28.6 (0.49)	6.9 (0.12)	199.8 (3.40)	6.1 (0.10)	1.6 (0.02)	0.9 (0.01)	0.5 (0.01)
KCSC B	3800.0 (95.80)	18.7 (0.47)	6.1 (0.15)	136.7 (3.46)	3.3 (0.08)	0.9 (0.02)	0.7 (0.01)	0.4 (0.01)

The values in parentheses indicate the relative content of each alkaloid to total amount of alkaloids analyzed in each KCSC.

**Table 2:** Accumulative changes in ambulatory, rearing, and rotational activities at P1, P2, and P3 after repeated administration of vehicle, nicotine, KCSC A, and KCSC B.

Groups	A. Ambulatory Activity								
	0–20 Min			20–40 Min			40–60 Min		
14 days of repeated vehicle	850.00	±	180.93	151.67	±	118.77	30.67	±	8.08
14 days of repeated nicotine	3840.50	±	451.28 ^#^	997.33	±	228.43 ^#^	773.67	±	305.43
14 days of repeated KCSC A	4124.50	±	629.04 *	1861.83	±	298.11 *^,+^	1993.00	±	344.85 *^,+^
14 days of repeated KCSC B	4641.67	±	561.08 *	1578.33	±	159.52 *	1452.83	±	64.61 *^,+^
**Groups**	**B. Rearing Activity**								
	**0–20 Min**			**20–40 Min**			**40–60 Min**		
14 days of repeated vehicle	210.83	±	70.36	22.83	±	20.27	7.00	±	1.73
14 days of repeated nicotine	952.17	±	131.06 ^#^	299.83	±	71.24 ^#^	191.67	±	76.22
14 days of repeated KCSC A	1052.00	±	156.76 *	612.33	±	93.19 *^,+^	667.33	±	111.59 *^,+^
14 days of repeated KCSC B	905.50	±	170.71 *	460.67	±	69.61 *	445.17	±	26.70 *^,+^
**Groups**	**C. Rotational Activity**								
	**0–20 Min**			**20–40 Min**			**40–60 Min**		
14 days of repeated vehicle	24.17	±	5.63	6.00	±	3.81	2.50	±	0.56
14 days of repeated nicotine	96.83	±	6.89 ^#^	41.00	±	7.29 ^#^	26.33	±	9.63
14 days of repeated KCSC A	107.33	±	11.25 *	68.17	±	9.20 *^,+^	68.33	±	7.83 *^,+^
14 days of repeated KCSC B	121.00	±	12.21 *	51.67	±	6.12 *	48.17	±	1.14 *^,+^

Statistical significance was determined by two-way RM-ANOVA and multiple comparison test with Bonferroni’s post-hoc test. ^#^ *p* < 0.05, repeated vehicle group vs. repeated nicotine group; * *p* < 0.05, repeated vehicle group vs. repeated KCSC groups; ^+^ *p* < 0.05, repeated nicotine group vs. repeated KCSC groups. *n* = 6 per group.

**Table 3:** Accumulative changes in ambulatory, rearing, and rotational activities at P1, P2, and P3 after challenge administration of vehicle, nicotine, KCSC A, and KCSC B.

Groups	A. Ambulatory Activity								
	P1 (0–20 Min)			P2 (20–40 Min)			P3 (40–60 Min)		
Vehicle challenge	550.33	±	118.12	137.83	±	108.50	54.83	±	23.88
Nicotine challenge	3510.50	±	468.54 ^#^	1512.83	±	337.40 ^#^	1037.33	±	299.56
KCSC A challenge	4305.33	±	634.05 *	2471.33	±	547.58 *^,+^	2496.00	±	544.72 *^,+^
KCSC B challenge	4483.50	±	446.57 *	1986.00	±	376.23 *	1693.33	±	293.24 *^,+^
**Groups**	**B. Rearing Activity**								
	**P1 (0–20 Min)**			**P2 (20–40 Min)**			**P3 (40–60 Min)**		
Vehicle challenge	254.00	±	80.47	23.67	±	20.16	12.00	±	6.91
Nicotine challenge	1003.50	±	190.27 ^#^	486.50	±	107.50^#^	291.83	±	74.58
KCSC A challenge	1069.67	±	136.39 *	879.33	±	154.75*^,+^	828.00	±	162.69 *^,+^
KCSC B challenge	1051.17	±	148.83 *	626.67	±	133.66*	532.67	±	101.85 *^,+^
**Groups**	**C. Rotational Activity**								
	**P1 (0–20 Min)**			**P2 (20–40 Min)**			**P3 (40–60 Min)**		
Vehicle challenge	22.33	±	3.71	4.00	±	2.25	2.83	±	0.70
Nicotine challenge	89.83	±	6.72 ^#^	51.83	±	11.03 ^#^	34.67	±	9.44
KCSC A challenge	109.50	±	10.92 *	73.83	±	9.78 *^,+^	74.83	±	11.48 *^,+^
KCSC B challenge	123.17	±	8.60 *	66.17	±	10.20 *	51.17	±	6.58 *^,+^

Statistical significance was determined by two-way RM-ANOVA and multiple comparison test with Bonferroni’s post-hoc test. ^#^ *p* < 0.05, vehicle challenge group vs. nicotine challenge group; * *p* < 0.05, vehicle challenge group vs. KCSC challenge groups; ^+^ *p* < 0.05, nicotine challenge group vs. KCSC challenge groups. *n* = 6 per group.

**Table 4:** Mean glutamate concentrations ([Glu]) and rate of changes in these concentrations (Δ[Glu]) in the dorsal striatum at three different time periods after 14 days repeated administrations of vehicle, nicotine, KCSC A, and KCSC B.

Groups	Mean Of [Glu] (Nm)								
	0–20 Min			20–40 Min			40–60 Min		
14 days of repeated vehicle	−38.76	±	30.12	−208.02	±	56.44	−311.49	±	110.71
14 days of repeated nicotine	153.70	±	39.56 ^#^	−35.41	±	52.93	−317.70	±	113.70
14 days of repeated KCSC A	470.13	±	42.72 *^,+^	608.43	±	92.48 *^,+^	455.21	±	92.60 *^,+^
14 days of repeated KCSC B	346.11	±	32.57 *^,+^	485.59	±	73.82 *	414.44	±	91.82 *^,+^
**Groups**	**Δ** **[Glu] (Nm)**								
	**0–20 Min**			**20–40 Min**			**40–60 Min**		
14 days of repeated vehicle	−38.76	±	30.12	−169.25	±	67.53	−103.47	±	55.11
14 days of repeated nicotine	153.70	±	39.56 ^#^	−189.11	±	71.60	−282.30	±	80.12
14 days of repeated KCSC A	470.13	±	42.72 *^,+^	133.30	±	51.65 *^,+^	−153.22	±	38.52
14 days of repeated KCSC B	346.11	±	32.57 *^,+^	139.47	±	45.10 *^,+^	−71.15	±	48.66

Statistical significance was determined by two-way RM-ANOVA and multiple comparison test with Bonferroni’s post-hoc test. ^#^ *p* < 0.05, repeated vehicle group vs. repeated nicotine group; * *p* < 0.05, repeated vehicle group vs. repeated KCSC groups; ^+^ *p* < 0.05, repeated nicotine group vs. repeated KCSC groups. *n* = 6 per group.

## Data Availability

Data is available on request.

## Supplementary Materials

The following are available online at https://www.mdpi.com/2076-3425/11/1/14/s1. Figure S1, Timelines for open-field test and glutamate biosensing; Figure S2, Implantation and placements of a glutamate biosensor in the dorsal striatum; Figure S3, Relative ratios of time spent in behavioral states after the 14 days repeated administration of KCSCs; Figure S4, Relative ratios of time spent in behavioral states after the challenge administration of KCSCs; Figure S5, In vitro current-time and calibration plots; Supplementary; Table S1, Accumulative changes in ambulatory, stereotypy, and resting time after 14 days repeated ad-ministration of KCSCs; Table S2, Accumulative changes in ambulatory, stereotypy, and resting time after the challenge administration of KCSCs.
