# Supplementary material for: Exposure to Commercial Cigarette Smoke Produces Psychomotor Sensitization via Hyperstimulation of Glutamate Response in the Dorsal Striatum
Source: Brain Sci. 2020 Dec 24;11(1):14. doi: 10.3390/brainsci11010014 (PMC7830476; doi:10.3390/brainsci11010014)
Supplement: Supplementary file 1 [file brainsci-11-00014-s001.pdf]

brain sciences

Supplementary Materials

Exposure to commercial cigarette smoke produces psychomotor sensitization via hyperstimulation of glutamate response in the dorsal striatum

In Soo Ryu[1,2,*], Jieun Kim[1], Ju Hwan Yang[1], Su Yeon Seo[1,4], Sumin Sohn[1], Sunghyun Kim[1], Kyuhong Lee[3], Joung-Wook Seo[2], Eun Sang Choe[1,*]

[1] Department of Biological Sciences, Pusan National University, 63-2 Busandaehak-ro, Geumjeong-gu, Busan 46241, Republic of Korea
[2] Research Center for Convergence Toxicology, Korea Institute of Toxicology, 141 Gajeong-ro, Yuseong-gu, Daejeon 34114, Republic of Korea
[3] Inhalation Toxicology Research Center, Korea Institute of Toxicology, 30 Baekhak 1-gil, Jeongeup 56212, Republic of Korea
[4] Korean Medicine Fundamental Research Division, Korea Institute of Oriental Medicine, 1672 Yuseong-daero, Yuseong-gu, Daejeon 34054, Republic of Korea

* Correspondence: insoo.ryu@kitox.re.kr, iaminsooryu@gmail.com (I.S.R.); eschoe@pusan.ac.kr (E.S.C.)
Tel.: +82-42-610-8293 (I.S.R.); +82-51-510-2272 (E.S.C.)

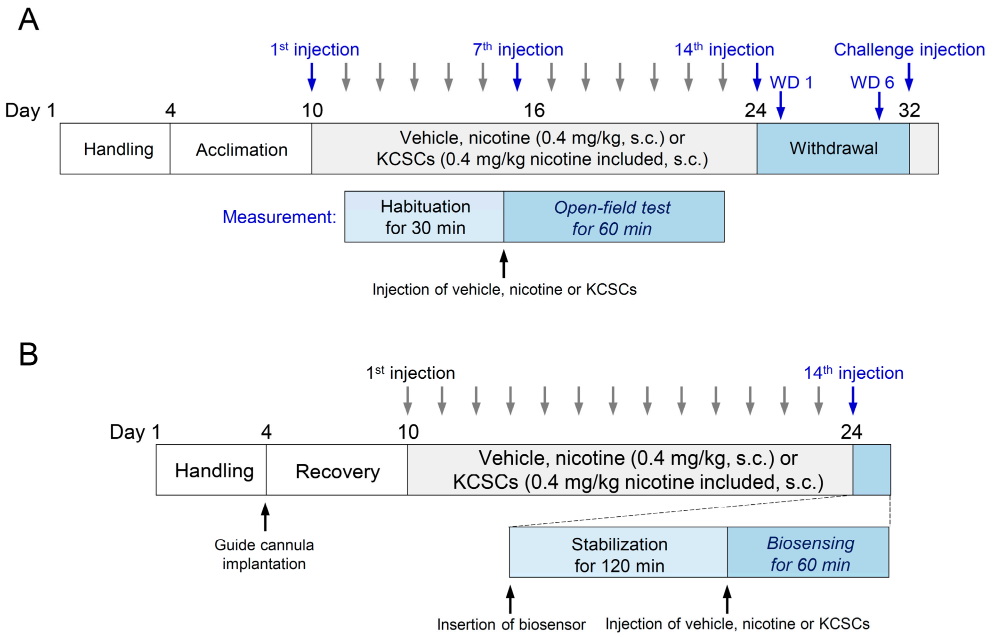

Supplementary Figure S1. Timelines for open-field test (A) and glutamate biosensing (B).

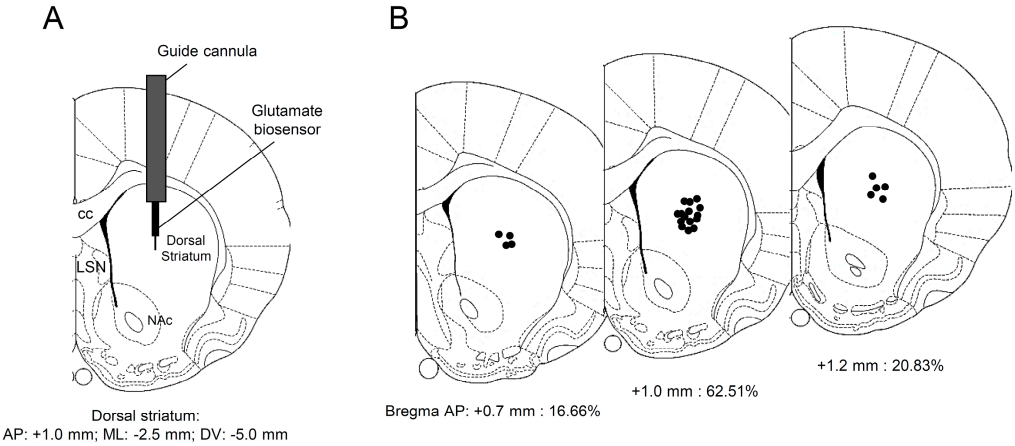

Supplementary Figure S2. Implantation (A) and placements (B) of a glutamate biosensor in the center of dorsal striatum. AP, anterior to the bregma; ML, right of the midline; DV, below the surface of the skull; cc, corpus callosum; LSN, lateral septic nucleus; NAc, nucleus accumbens.

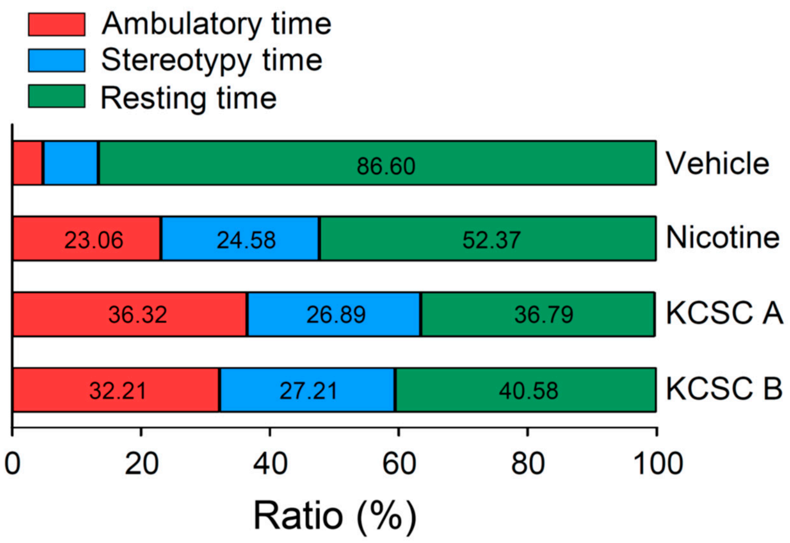

Supplementary Figure S3. Relative ratios of time spent in behavioral states after the 14th administration of vehicle, nicotine, KCSC A, and KCSC B. The number in each bar represents relative percentage of each behavioral state during a 60 min test session.

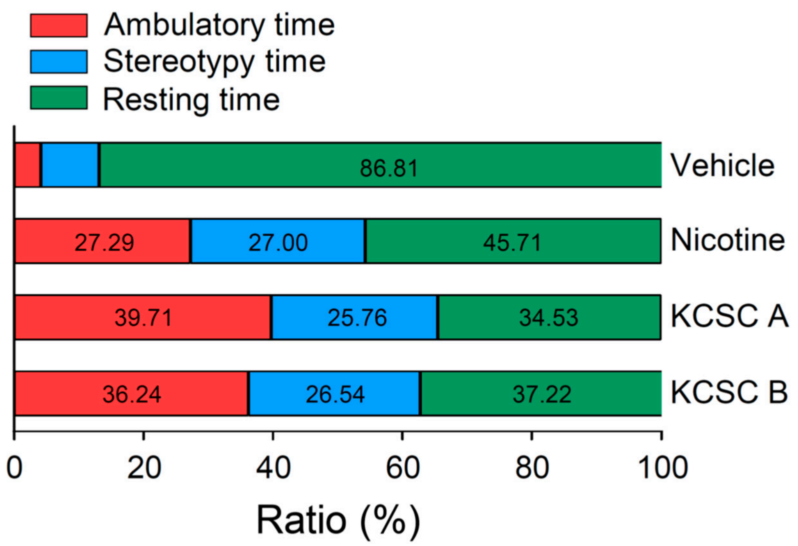

Supplementary Figure S4. Relative ratios of time spent in behavioral states after the challenge administration of vehicle, nicotine, KCSC A, and KCSC B. The number in each bar represents relative percentage of each behavioral state during a 60 min test session.

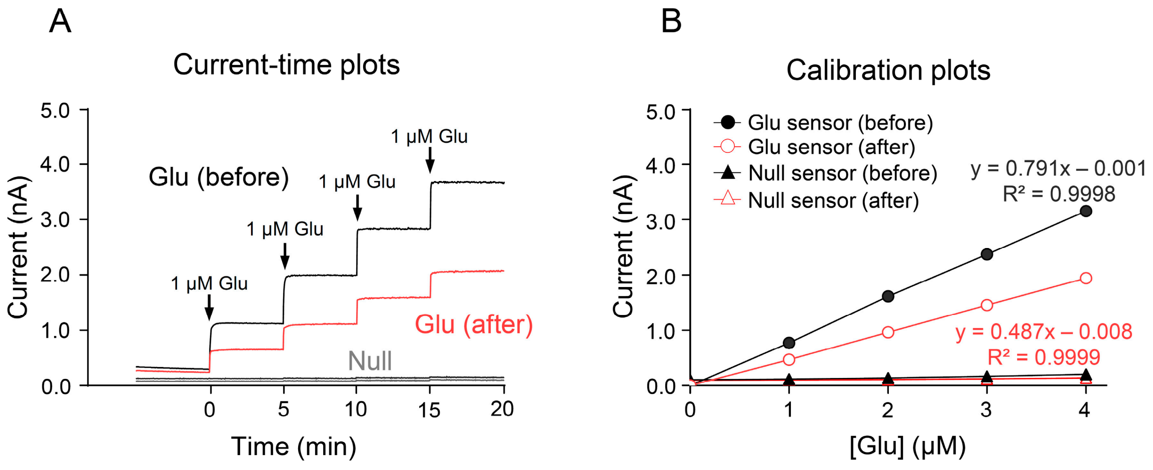

Supplementary Figure S5. In vitro current-time (A) and calibration (B) plots. [Glu], glutamate concentration.

Supplementary Table S1. Accumulative changes in ambulatory, stereotypy, and resting time at P1, P2, and P3 after 14 days repeated administration of vehicle, nicotine, KCSC A and KCSC B

Groups	A. Ambulatory time								
	0 – 20 min			20 – 40 min			40 – 60 min		
14 days of repeated vehicle	131.83	±	33.05	30.33	±	19.82	9.67	±	2.33
14 days of repeated nicotine	510.50	±	$38.59^{\#}$	227.83	±	$34.56^{\#}$	150.00	±	$41.81^{\#}$
14 days of repeated KCSC A	604.33	±	16.15^{*}	343.50	±	44.23^{*}	363.17	±	$43.67^{*,+}$
14 days of repeated KCSC B	607.33	±	32.07^{*}	288.00	±	28.79^{*}	264.33	±	$11.85^{*,+}$
**Groups**	**B. Stereotypy time**								
	0 – 20 min			20 – 40 min			40 – 60 min		
14 days of repeated vehicle	210.83	±	26.19	69.17	±	33.18	48.17	±	15.25
14 days of repeated nicotine	952.17	±	$15.64^{\#}$	355.17	±	$40.16^{\#}$	238.17	±	$52.32^{\#}$
14 days of repeated KCSC A	1052.00	±	23.84^{*}	349.83	±	8.06^{*}	319.67	±	10.80^{*}
14 days of repeated KCSC B	905.50	±	9.45^{*}	344.33	±	29.55^{*}	354.17	±	30.51^{*}
**Groups**	**C. Resting time**								
	0 – 20 min			20 – 40 min			40 – 60 min		
14 days of repeated vehicle	908.67	±	49.33	1099.83	±	52.67	1116.17	±	17.75
14 days of repeated nicotine	343.50	±	$29.94^{\#}$	617.00	±	$68.92^{\#}$	811.83	±	$93.41^{\#}$
14 days of repeated KCSC A	352.67	±	82.23^{*}	538.83	±	71.54^{*}	549.00	±	$61.07^{*,+}$
14 days of repeated KCSC B	311.50	±	30.90^{*}	567.67	±	52.00^{*}	581.50	±	$33.64^{*,+}$

Statistical significance was determined by two-way RM-ANOVA and multiple comparison test with Bonferroni's post-hoc test. $^{\#}p<0.05$, repeated vehicle group vs. repeated nicotine group; $^{*}p<0.05$, repeated vehicle group vs. repeated KCSC groups; $^{+}p<0.05$, repeated nicotine group vs. repeated KCSC groups. n=6 per group.

Supplementary Table S2. Accumulative changes in ambulatory, stereotypy, and resting time at P1, P2, and P3 after the challenge administration of vehicle, nicotine, KCSC A and KCSC B

Groups	A. Ambulatory time		
	0 – 20 min	20 – 40 min	40 – 60 min
Vehicle challenge	109.33 ± 23.16	27.17 ± 19.73	13.67 ± 4.09
Nicotine challenge	496.50 ± 27.38#	285.83 ± 64.95#	200.17 ± 59.18#
KCSC A challenge	611.00 ± 37.24*,+	405.50 ± 53.08*	413.17 ± 63.14*,+
KCSC B challenge	645.50 ± 40.87*,+	345.50 ± 57.57*	313.67 ± 41.36*
**Groups**	**B. Stereotypy time**		
	0 – 20 min	20 – 40 min	40 – 60 min
Vehicle challenge	178.67 ± 28.36	51.83 ± 23.39	35.83 ± 6.11
Nicotine challenge	350.83 ± 11.77#	348.33 ± 25.82#	272.83 ± 65.33#
KCSC A challenge	283.50 ± 24.62*	318.17 ± 16.96*	325.83 ± 18.62*
KCSC B challenge	273.17 ± 12.34*	332.67 ± 25.43*	349.67 ± 31.67*
**Groups**	**C. Resting time**		
	0 – 20 min	20 – 40 min	40 – 60 min
Vehicle challenge	911.83 ± 64.42	1121.00 ± 43.06	1150.50 ± 9.41
Nicotine challenge	352.67 ± 22.96#	565.83 ± 83.72#	727.00 ± 114.59#
KCSC A challenge	305.50 ± 24.90*	476.33 ± 48.37*	455.17 ± 44.14*,+
KCSC B challenge	294.83 ± 17.63*	512.83 ± 74.48*	538.50 ± 49.47*

Statistical significance was determined by two-way RM-ANOVA and multiple comparison test with Bonferroni's post-hoc test. #$p<0.05$, vehicle challenge group vs. nicotine challenge group; *$p<0.05$, vehicle challenge group vs. KCSC challenge groups; +$p<0.05$, nicotine challenge group vs. KCSC challenge groups. n=6 per group.
